# Cerebrospinal Fluid-Derived Microvesicles From Sleeping Sickness Patients Alter Protein Expression in Human Astrocytes

**DOI:** 10.3389/fcimb.2019.00391

**Published:** 2019-11-20

**Authors:** Vito Dozio, Veerle Lejon, Dieudonné Mumba Ngoyi, Philippe Büscher, Jean-Charles Sanchez, Natalia Tiberti

**Affiliations:** ^1^Translational Biomarker Group, University of Geneva, Geneva, Switzerland; ^2^Intertryp, Institut de Recherche pour le Développement, CIRAD, University of Montpellier, Montpellier, France; ^3^Department of Parasitology, Institut National de Recherche Biomédicale, Kinshasa, Democratic Republic of Congo; ^4^Department of Biomedical Sciences, Institute of Tropical Medicine, Antwerp, Belgium; ^5^Department of Infectious - Tropical Diseases and Microbiology, IRCCS Sacro Cuore Don Calabria Hospital, Verona, Italy

**Keywords:** human African trypanosomiasis, microvesicles, cerebrospinal fluid, DIA-MS, astrocytes

## Abstract

Human African trypanosomiasis (HAT) caused by the extracellular protozoon *Trypanosoma brucei*, is a neglected tropical disease affecting the poorest communities in sub-Saharan Africa. HAT progresses from a hemolymphatic first stage (S1) to a meningo-encephalitic late stage (S2) when parasites reach the central nervous system (CNS), although the existence of an intermediate stage (Int.) has also been proposed. The pathophysiological mechanisms associated with the development of S2 encephalopathy are yet to be fully elucidated. Here we hypothesized that HAT progression toward S2 might be accompanied by an increased release of microvesicles (MVs), sub-micron elements (0.1–1 μm) involved in inflammatory processes and in the determination of the outcome of infections. We studied the morphology of MVs isolated from HAT cerebrospinal fluid (CSF) by transmission electron microscopy (TEM) and used flow cytometry to show that total-MVs and leukocyte derived-CD45^+^ MVs are significantly increased in concentration in S2 patients' CSF compared to S1 and Int. samples (*n* = 12 per group). To assess potential biological properties of these MVs, immortalized human astrocytes were exposed, *in vitro*, to MVs enriched from S1, Int. or S2 CSF. Data-independent acquisition mass spectrometry analyses showed that S2 MVs induced, compared to Int. or S1 MVs, a strong proteome modulation in astrocytes that resembled the one produced by IFN-γ, a key molecule in HAT pathogenesis. Our results indicate that HAT S2 CSF harbors MVs potentially involved in the mechanisms of pathology associated with HAT late stage. Such vesicles might thus represent a new player to consider in future functional studies.

## Introduction

Human African trypanosomiasis (HAT, sleeping sickness) is a vector-borne neglected tropical disease caused by the extracellular protozoon *Trypanosoma brucei*. The parasite is limited to sub-Saharan Africa, where it is transmitted by the tse-tse fly (Buscher et al., 2017). Since the beginning of this century, a progressive decrease in the number of HAT reported cases has been observed and, in 2016, 2,164 new cases were reported (Franco et al., 2018), in line with the WHO objective of HAT elimination as a public health problem by 2020 (Maurice, 2013). Nonetheless, a number of relevant issues, including the presence of animal reservoirs and of asymptomatic cases bearing extravascular parasites, still represent potential threats for a possible HAT re-emergence (Buscher et al., 2017).

Two trypanosome sub-species—*T. b. gambiense* and *T. b. rhodesiense*—are responsible for two geographically separated and clinically different forms of disease. *T. b. gambiens*e (Central and Western Africa) causes a chronic infection, covering ~97% of all HAT cases, while *T. b. rhodesiense* (Eastern Africa) is responsible for an acute disease (Miezan et al., 2000). In both cases, the disease progresses from a first stage (S1, early) characterized by peripheral parasitemia, to a second stage (S2, late) when parasites reach the central nervous system (CNS) (Buscher et al., 2017). Stage determination is based on cerebrospinal fluid (CSF) examinations for white blood cell (WBC) counting and parasite detection (WHO, 2013).

The pathophysiological mechanisms associated with disease progression and the development of the neuro-inflammatory reaction that accompanies HAT late stage are still to be fully elucidated. Numerous pieces of evidence have, however, been collected through studies in animal models and “omics investigations of patients” biological fluids (Kristensson et al., 2010; Tiberti et al., 2010, 2015; Masocha, 2013). Indeed, the infiltration of immune cells, activation of astrocytes and microglia, and the release of pro-inflammatory cytokines have been well-documented (Kennedy and Rodgers, 2019). To the best of our knowledge, the potential involvement of extracellular vesicles (EVs) within this scenario is yet to be investigated. EVs are sub-micron vesicular structures released by potentially all cell types, including microorganisms (Schorey and Harding, 2016). Two different populations of EVs are of particular biological and clinical interest: (i) exosomes (EXOs, 40–100 nm)—originating from the endocytic pathway; and (ii) microvesicles (MVs, 0.1–1 μm)—originating by membrane budding (Raposo and Stoorvogel, 2013). EVs are now recognized for representing an important mechanism of intercellular communication, thanks to their ability to deliver their cargo of proteins, nucleic acids, and metabolites from donor to recipient cells (Maas et al., 2017). Importantly, EVs have been shown to participate in the host-pathogen interaction as well as in the cross-talk between parasites in a number of parasitic diseases (Coakley et al., 2015; Schorey and Harding, 2016). Here, we hypothesized that the neuro-inflammation that characterizes HAT late stage might be accompanied by an increased release of extracellular vesicles. To verify our hypothesis, we evaluated the presence of host-derived MVs in the CSF of HAT patients and assessed their potential effects, at the protein level, on human astrocytes *in vitro*.

## Methods

### Population Description and Sample Collection

CSF samples were obtained from *T. b. gambiense* HAT patients, enrolled in the Democratic Republic of the Congo in the context of a longitudinal study for the post-therapeutic outcome evaluation approved by the Ministry of Health of the Democratic Republic of the Congo and by the Commission for Medical Ethics of the Institute of Tropical Medicine Antwerp, Belgium (Mumba Ngoyi et al., 2010). All patients (or a relative/guardian) whose samples have been analyzed in the present study had signed a written informed consent. HAT stage was established according to WHO guidelines, i.e., S1 if CSF WBC ≤ 5/μL and absence of parasites; S2 if CSF WBC > 5/μL and/or presence of trypanosomes (WHO, 2013). For the present study, S2 samples having either a low WBC ≤ 5/μL and parasites in CSF, or absence of parasites and WBC count between 6 and 20/μL were considered as intermediate stage (Int.), as already reported elsewhere (Doua et al., 1996; Lejon et al., 2003). The number of WBC in CSF (WBC/μl) was determined manually by trained operators using disposable counting chambers (Uriglass; Menarini) (Mumba Ngoyi et al., 2010). CSF samples were then subjected to the modified single centrifugation for parasite detection (Miezan et al., 2000), and the supernatant of this centrifugation, considered free of cells, was aliquoted and stored at −80°C until further use. All CSF samples here analyzed were thus kept at −80°C and submitted to the same experimental conditions.

### MV Enrichment and Transmission Electron Microscopy

CSF samples were centrifuged 5 min at 2,000 g to remove possible large debris and MVs were subsequently enriched by repeated centrifugations at 18,000 g for 50 min with PBS washes between centrifugations (Tiberti et al., 2016). MV pellet was visualized by transmission electron microscopy (TEM), following a protocol adapted from Latham et al. (2015). Briefly, 6 μl of MVs enriched from 3 ml of CSF were absorbed onto ultra-thin carbon coated-copper grids (200 mesh, Electron Microscopy Sciences), blocked with 1% BSA in PBS (w/v) and labeled with 5 μg/ml biotin-Annexin V (BD Biosciences). Grids were fixed with 2% paraformaldehyde, quenched with 0.05 M glycine prior to incubation with streptavidin-gold 10 nm (Aurion) 1:25 in 0.1% BSA. Grids were post-fixed with 2% glutaraldehyde and negative stained with 2% uranyl acetate. TEM images were acquired on a Tecnai™ G2 Sphera electron microscope (FEI, Eindhoven) at 120 kV and analyzed using Fiji (ImageJ) (Schindelin et al., 2012).

### Flow Cytometry

MVs from S1, Int. and S2 patients' CSF (*n* = 12 per group, 20 μl per sample) were enumerated and phenotyped by flow cytometry (Table S1). Annexin V-FITC (Beckman Coulter) and CellTrace™ Violet reagent (CTV—ThermoFisher Scientific) were used to detect both phosphatidylserine (PS) positive and negative MVs. To establish MV cellular origin, the following markers were detected using specific monoclonal antibodies: CD45 for leukocyte-derived MVs (L-MVs), CD105 for endothelial cell-derived MVs (E-MVs), and neuron specific enolase (NSE) for neuron-derived MVs (N-MVs) (Table S2, Figure S1). Two multicolor combinations were established to label 20 μl of CSF: (I) annexin V-FITC, CTV, anti-CD105-PE, anti-CD45-APC; (II) annexin V-FITC, CTV, anti-NSE-PE. Samples were acquired on a Gallios flow cytometer (Beckman Coulter) over 10 min at a low flow rate, with particle detection triggered on forward scatter (FS) and set to target submicron particle sizes. Compensation matrices were established using VersaComp Antibody Capture Beads (Beckman Coulter) while Flow-Count Fluorospheres (Beckman Coulter, lot concentration 1,006/μl) were added to each assay to determine MVs concentration (MV/μl of CSF) according to manufacturer's instructions. Unlabeled CSF and CSF labeled using isotype control antibodies (Table S2) were also analyzed to account for CSF potential auto-fluorescence and antibody unspecific binding, respectively. Data were analyzed with Kaluza Analysis software v2.1 (Beckman Coulter).

Statistical analyses were performed with STATA v14.0 (StataCorp LLC). Kruskal–Wallis test, followed by Bonferroni correction, was used to compare MV concentration among the three HAT groups with significance set at 5%. Univariate linear regression was used to assess the association between MV concentration and demographic or clinical parameters. Predictors showing a *p* < 0.1 were further tested in a multivariate model.

### Astrocytes Exposure to MVs

Immortalized human astrocytes (fetal—SV40, Applied Biological Material Inc.) were seeded (10,000 cells/well) in 96-well plates coated with 0.01 mg/ml collagen type I—rat tail (Merck KGaA) and maintained in Prigrow IV Medium (Applied Biological Material Inc.) supplemented with 5% heat-inactivated fetal bovine serum (FBS, Gibco), 100 U/ml penicillin, 100 μg/ml streptomycin and grown until confluent at 37°C in 5% CO_2_.

Confluent cells were exposed to MVs in FBS-free medium for 24 h at 37°C in 5% CO_2_. MVs were enriched from 2.4 ml of S1 CSF (*n* = 2), Int. CSF (*n* = 2), or S2 CSF (*n* = 3), obtained by pooling samples from gender and stage matched patients (Table S3). Cells exposed to IFN-γ (0.5 U/μl, *n* = 3) for 24 h were used as a pro-inflammatory control, while cells incubated with FBS-free medium only (*n* = 3) as untreated control. Cytotoxicity was tested via LDH activity (ThermoScientific). The same design was followed when exposing astrocytes to MV-free CSF diluted 1:10 in FBS-free medium.

### Protein Digestion and Data Independent Acquisition Mass Spectrometry (DIA-MS) Analyses

Astrocyte proteins were extracted with 0.1% RapiGest SF (Waters) in 0.1 M triethylammonium bicarbonate buffer (TEAB), pH 8.0. Samples were sonicated, incubated for 10 min at 80°C, spun (14,000 g, 10 min) and finally the supernatant was recovered. The protein concentration was then determined using the Bradford protein assay. For each sample, 9 μg of proteins were reduced with 5 mM Tris (2-carboxyethyl)phosphine hydrochloride (TCEP), alkylated with 15 mM iodoacetamide and digested with sequencing grade-modified trypsin (Promega, 1:50 protease to protein ratio). The peptide mixture was then desalted with C18 spin columns (Harvard apparatus), dried under vacuum, re-suspended in 5% acetonitrile, 0.1% formic acid (peptide final concentration of 0.5 μg/μL), and spiked with iRT peptides (Biognosys) 1:20.

The equivalent of 2 μg of peptides were analyzed using Liquid Chromatography–Electrospray Ionization–MS/MS (LC-ESI-MS/MS) on an Orbitrap Fusion Lumos Tribrid mass spectrometer (Thermo Fisher Scientific) equipped with a Thermo EASY-nLC following a published protocol (Dozio and Sanchez, 2018). Briefly, for generating the spectral library full lysates from all samples together with 12 OFFgel fractionated samples were first acquired in data dependent acquisition mode (DDA) and analyzed with Proteome Discoverer 2.0 (Thermo Scientific) and the Mascot search engine. The search parameters included 10 ppm precursor mass tolerance, 0.02 Da fragment mass tolerance and two miscleavages. Static modifications included carbamidomethylation (+57.021 Da) on cysteines, whereas dynamic modifications included oxidation (+15.995 Da) on methionines. Peptide spectrum matches (PSMs) were verified using the Percolator module (FDR < 0.01). Proteome Discoverer results were then imported in Spectronaut (Biognosys) and used to generate a spectral library as previously reported (Dozio and Sanchez, 2018). The 16 samples corresponding to the different study conditions (**Figure 2A**) plus a pool of them injected three times over the whole MS analysis to assess the technical variability, were then acquired in DIA mode (Dozio and Sanchez, 2018) and the raw DIA MS data matched against the spectral library using Spectronaut. Peptide intensities were finally exported and analyzed using mapDIA (Teo et al., 2015).

For each condition, a protein fold change (FC) was computed with respect to untreated control cells; proteins with FDR < 0.05 and absolute |FC| > 1.2 were considered significantly differentially abundant (Dozio and Sanchez, 2018). The list of astrocyte proteins differential in at least one study condition was then analyzed with MetaCore™ (Clarivate Analytics) to highlight significantly represented process networks, i.e., molecular and cellular processes representing pre-defined network of protein interactions characteristic of each process (Bessarabova et al., 2012).

The mass spectrometry proteomics data have been deposited to the ProteomeXchange Consortium via the PRIDE (Perez-Riverol et al., 2019) partner repository with the dataset identifier PXD014945.

## Results

**Figure 1:**
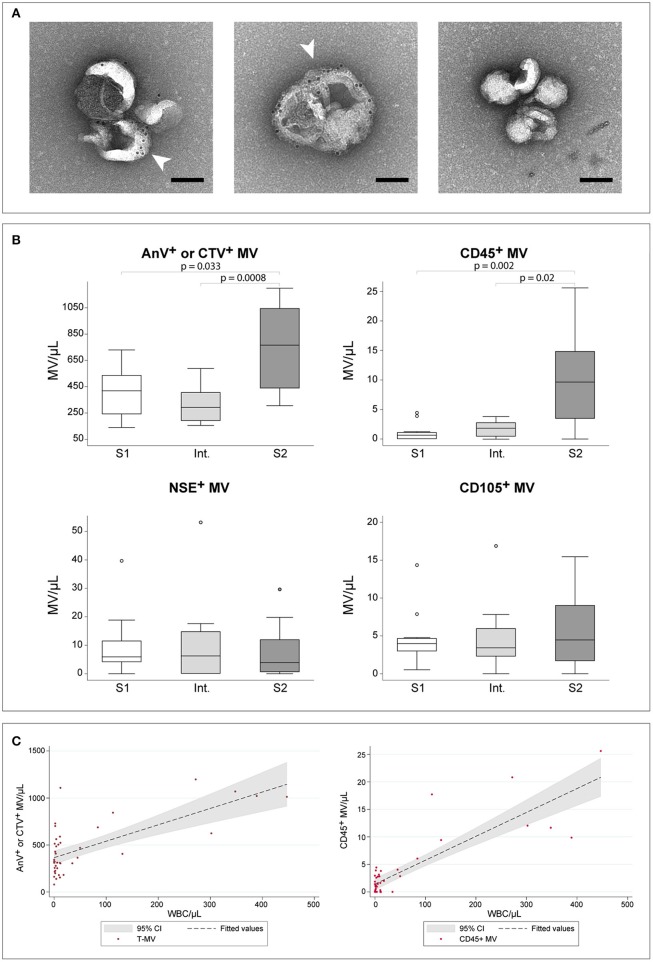
HAT CSF-derived MVs. **(A)** Immuno-gold labeled MVs derived from CSF and visualized by TEM at 25000X magnification. White arrow-heads indicate phosphatydyl serine labeling with biotin-AnnexinV and 10 nm gold-streptavidin. The right panel represents the negative control prepared without AnnexinV. Scale bar, 100 nm. **(B)** Comparison of MVs concentration in CSF as established by flow cytometry. Comparisons were assessed using the Kruskal–Wallis test, followed by Bonferroni correction. Only significant *p*-values are reported. For each group, *n* = 12. **(C)** Scatter plots showing the linear relation between WBC/μl and the concentration of total-MVs (left) or of CD45^+^ leukocyte-derived MVs (right). The regression line and the 95% confidence interval are reported.

The visualization by TEM of CSF-derived MVs revealed the presence of vesicular elements with diameter ranging between 100 and 300 nm (Figure 1A). The presence of phosphatidylserine (PS) at the vesicle surface was confirmed by immuno-gold labeling using biotinylated-Annexin V and 10 nm gold-streptavidin (Figure 1A, white arrowheads). The CSF concentration of total MVs, determined as Annexin V^+^ or CTV^+^ elements by flow cytometry, resulted significantly increased in S2 samples when compared to both S1 and Int. samples (*p* = 0.033 and *p* = 0.0008, respectively) (Figure 1B). Similarly, the concentration of CD45^+^ L-MVs was significantly increased in S2 samples (*p* = 0.002 to S1, *p* = 0.02 to Int.). The two other sub-populations, i.e., CD105^+^ E-MVs and NSE^+^ N-MVs, did not show any differences in their concentration among the three groups of HAT samples.

Linear regression analyses testing WBC/μl, gender, age, presence of trypanosomes in the CSF and presence of neurological signs as independent predictors for the number of MVs revealed a significant relationship between total-MV/μl and either WBC/μl (*p* < 0.0001, *R*^2^ = 0.477) or presence of trypanosomes in CSF (*p* = 0.001, R^2^ = 0.263). The multivariate model, however, did not increase the performance of WBC, which alone showed a highly significant linear relationship with total-MVs/μl (Table 1A, Figure 1C). Similar results were obtained for L-MV/μl (Figure 1C). Indeed, univariate analyses showed a significant relationship with WBC count (WBC/μl) (*p* < 0.0001, *R*^2^ = 0.723), presence of parasites in the CSF (*p* < 0.0001, *R*^2^ = 0.342) and patients' gender (*p* = 0.028, *R*^2^ = 0.135), with WBC/μl being the best predictor (Table 1B). Interestingly, WBC alone showed better performances than the disease stage in predicting both total-MVs and leukocyte-derived MV concentrations (Table S4). No association between N-MVs/μl or E-MVs/μl and any of the tested variables was observed (Table S4).

**Table 1:** Regression analysis assessing the association between total-MVs (T-MVs) **(A)** or CD45^+^ L-MV **(B)** and demographical and clinical variables.

**Variables**	**Intercept**	**[95% Confidence interval]**		***p*-value**	**Model R-squared**
**A—REGRESSION ANALYSIS FOR T-MVs**					
**Univariate**					
WBC/μL	1.67	1.06	2.28	<0.0001	0.477
Sex (M)	152.84	−43.88	349.56	0.124	0.068
Age	−4.29	−11.77	3.19	0.252	0.038
T+ (yes)	307.52	128.05	486.98	0.001	0.263
NS+ (yes)	97.01	−109.26	303.29	0.346	0.026
Multivariate^*^					
WBC	1.46	0.68	2.24	0.001	0.457
T+ (Yes)	83.05	−110.42	276.51	0.389	
**B—REGRESSION ANALYSIS FOR CD45**^**+**^ **L-MVs**					
**Univariate**					
WBC/μL	0.04	0.03	0.05	<0.0001	0.723
Sex (M)	4.53	0.53	8.53	0.028	0.135
Age	−0.06	−0.22	0.10	0.424	0.019
T+ (yes)	7.40	3.83	10.98	<0.0001	0.342
NS+ (yes)	3.32	−0.94	7.57	0.122	0.069
Multivariate^*^					
WBC/μL	0.04	0.02	0.05	<0.0001	0.706
T+ (yes)	0.54	−1.67	4.74	0.336	
Sex (M)	0.80	−1.88	3.48	0.548	

* *For multivariate analysis, the adjusted R-squared is reported*.

*T+, presence of trypanosomes in CSF; NS+, presence of neurological signs*.

**Figure 2:**
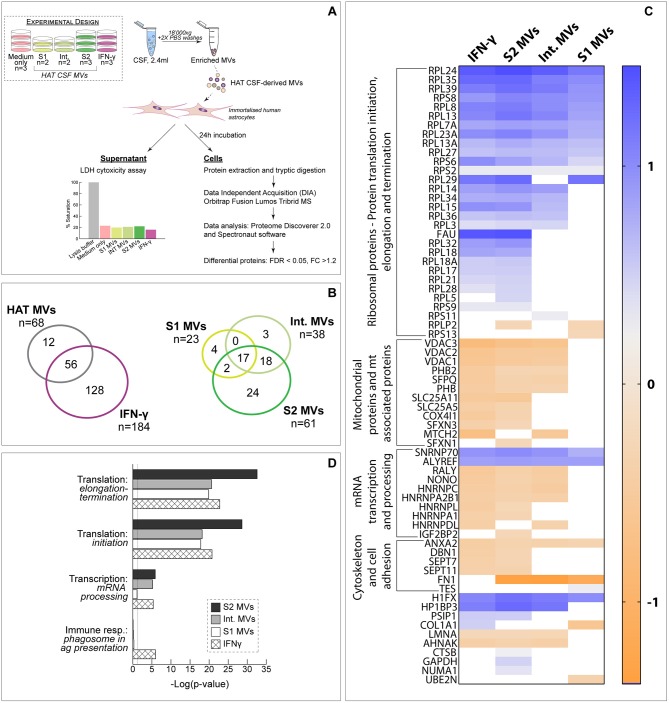
Exposure of human astrocytes to HAT CSF MVs. **(A)** Schematic representation of the experimental design. The results of the LDH assay, showing the absence of toxicity on astrocytes upon 24 h exposure to MVs, is also reported. **(B)** Comparison of the proteins identified in the different analyzed samples. **(C)** Heat-map comparing the protein fold change (FC) between the different study conditions and untreated control cells. Only proteins significantly differentially expressed (FDR < 0.05 and |FC| >1.2) upon exposure to at least one type of HAT MVs (i.e., S1, Int. or S2) are represented. **(D)** Process networks significantly represented amongst differentially abundant proteins.

To evaluate the potential pathogenic role of the vesicles, MVs enriched from CSF of patients with different stages of HAT (S1, Int., S2) were incubated with human astrocytes for 24 h and the alterations induced in cell protein abundance evaluated by DIA-MS. Cells were also incubated with IFN-γ as positive inflammatory control. First, it should be noted that MVs did not show any cytotoxic effect as determined by LDH assay (Figure 2A). DIA-MS analyses of astrocytes resulted in the identification and quantification of 17,891 peptides corresponding to 3,455 proteins with high reproducibility (averaged Pearson correlation coefficient of 0.98 for technical replicates) with no missing values (Supplementary Table 1). For each study condition, the protein FC was computed relative to untreated control cells. Overall, 196 proteins resulted significantly differentially abundant in at least one condition, 29% of which were commonly altered by HAT CSF-MVs and IFN-γ (Figure 2B). Among the three categories of HAT-MVs (i.e., derived from S1, Int. or S2 patients), cells exposed to S2-MVs showed the largest number of altered proteins (61 out of 68, i.e., 90%) while 17 proteins (25%) were commonly affected by the three types of HAT MVs (Figures 2B,C). Interestingly, all proteins significantly changing upon exposure to IFN-γ and S2-MVs had their abundance modulated with similar patterns, as shown in the heat-map (Figure 2C). Significantly changing proteins were then classified based on their biological function. Ribosomal proteins involved in protein translation resulted to be increased upon exposure to S2-MVs and IFN-γ and, to a lesser extent, to intermediate and S1 vesicles. On the other side, 12 mitochondrial or mitochondrial-associated proteins were decreased in abundance in both S2-MVs and IFN-γ treated cells compared to untreated control cells, seven of which were also altered upon exposure to Int. MVs but not to S1. Proteins associated with the cytoskeleton and cell adhesion were also decreased but mainly in astrocytes exposed to S2-MVs or to IFN-γ. Similar results were obtained upon performing a network analysis (Figure 2D). Four process networks were significantly represented amongst the proteins differentially expressed in the four study conditions. Interestingly, three out of the four were associated with mRNA processing or translation into proteins. The fourth process network, associated with the immune response, was significantly represented only amongst proteins altered upon IFN-γ exposure. These results were further confirmed by biological network analysis, which showed protein translation and cell organization to be affected by the exposure to HAT MVs (Table S5). To evaluate the potential presence of proteins more specifically associated with the disease progression, results were also analyzed comparing S1- or S2-MVs treated cells to Int.-MVs. Seven proteins were significantly altered in cells exposed to S1 MVs while none was differentially abundant in S2-exposed cells compared to Int (Tables S6, S7).

Less marked alterations in protein expression were observed when astrocytes were exposed to MV-free CSF, i.e., the supernatant of the centrifugation for MVs enrichment. In this case, overall 3,480 proteins were identified and quantified by DIA-MS, with an 82% overlap in protein identity with the experiment performed with MVs. Only 12 proteins resulted to be significantly changing upon exposure to MV-free HAT CSF and, among these, only four were altered by MV-free S2 CSF (Figure S2).

## Discussion

Microvesicles (MVs) are well-recognized molecular players involved in the intercellular communication in both physiological and pathological states (Schorey and Harding, 2016; Maas et al., 2017). Being considered an important source of disease biomarkers and a tool to better understand pathophysiological mechanisms, EVs derived from blood or blood components are currently being widely studied (Tissot et al., 2013). CSF is an important source of EVs as well (Patz et al., 2013; Welton et al., 2017), although less frequently investigated compared to blood. Nonetheless, the important association between vesicles and both neuro-inflammation and cellular communication within the brain compartment is now well-recognized (Fruhbeis et al., 2013; Delpech et al., 2019).

Here we showed, for the first time, that CSF derived from HAT patients infected with *T. b. gambiense* harbored MVs, the concentration of which was significantly increased in the CSF of late stage patients compared to both early and intermediate stages. Additionally, the phenotypic characterization of MVs based on their cellular origin, revealed that leukocyte-derived MVs expressing CD45 on their surface were significantly over-abundant in S2 CSF. Such a result is not surprising, given that the increased number of leukocytes in CSF is a well-known trait associated with HAT progression and the number of CSF WBC is used as a staging marker (Mumba Ngoyi et al., 2013; WHO, 2013). The increased concentration of L-MVs here observed is thus likely to reflect the activation state of leukocytes recruited to the brain compartment during late stage disease. The results obtained by regression analysis further support this hypothesis since a very strong association between the number of leukocytes per μl of CSF and both T-MVs and L-MVs was observed. Although, among the sub-populations here assessed, L-MVs showed the largest variations in concentration between HAT stages, it is possible that EVs originating from other cell types could contribute to the overall CSF-MV population (Fruhbeis et al., 2013). CSF MVs could also derive from the invading parasites as *T. brucei* was shown to shed vesicular elements in a few reports. Indeed, exosomal markers and vesicular-like structures were detected in the secretome of bloodstream *T. b. gambiense* (Geiger et al., 2010), and *T. brucei-*derived EVs were revealed to participate in the communication between parasites through the transfer of their content—including the serum-resistance associated (SRA) factor—(Szempruch et al., 2016) and in parasite migration and social motility (Eliaz et al., 2017). Nonetheless, the exact mechanisms of vesicular secretion in *T. brucei* are yet to be described.

Here, we also showed that MVs derived from S2 HAT patients' CSF display functional properties since, *in vitro*, they altered the protein expression of human astrocytes inducing changes similar to those observed in cells exposed to IFN-γ, a classical pro-inflammatory stimulus, which plays a central role, although still partly unclear, in HAT meningoencephalitis (Wu et al., 2017). Ribosomal proteins resulted to be significantly affected by the exposure to S2 MVs. These ubiquitous proteins play important roles in the process of ribosome assembly and transcript translation; their increased expression could thus be indicative of an increased protein synthesis consequent to cell activation. Nonetheless, since a number of years, it has been proposed that ribosomal proteins might also display extra-ribosomal functions and some of them are now considered as moonlight proteins, i.e., single proteins displaying multiple biochemical functions (Jeffery, 2015). Such proteins are gaining much attention for their potential importance in physiological and pathological conditions, as some have been associated with immune signaling (Zhou et al., 2015). Although a functional association between alteration in ribosomal proteins and astrocyte activation cannot be established yet, further verification of the results here obtained might contribute to elucidate the potential role of ribosomal proteins in neuro-inflammation. Additionally, several mitochondrial proteins were significantly decreased in abundance following exposure to S2 MVs and IFN-γ. These proteins regulate cell metabolism, apoptosis and reactive oxygen species (ROS) and their deficiency is associated with impairment of several cellular functions (Baghel and Thakur, 2019). We also found cytoskeletal proteins significantly decreased in their abundance, suggesting a potential dysregulation in cytoskeletal organization, after exposure to S2 MVs. Among this class of proteins, there were septins which are involved, among others, in actin dynamics and cell shape, but are also crucial during cytokinesis (Mostowy and Cossart, 2012).

MVs derived from intermediate and S1 CSF induced less marked alterations in astrocyte protein expression. This result could be related to the overall lower concentration of MVs compared to S2 CSF. However, it is worth noticing that, although we did not observe differences in concentration between S1 and Int. MVs, they showed a different impact on astrocyte protein expression when compared to controls. Indeed, the alterations in astrocyte protein abundances elicited by Int. MVs were “halfway” between those observed with S1 and S2 MVs. Nonetheless, only a very limited number of proteins was significantly altered in cells exposed to S1 MVs compared to Int. and none in S2 MVs compared to Int. suggesting that the response induced in target cells by MVs obtained from different HAT stages is not different in nature but rather in intensity. These observations further sustain the existence of an early second stage, in which parasites might already have crossed the BBB but the neuro-inflammatory response is not yet established and some patients might still be successfully treated with the S1 drug pentamidine (Doua et al., 1996).

HAT pathogenesis has yet to be elucidated, especially the mechanisms of disease progression that lead parasites to enter the CNS. A correlation between the increase in CNS IFN-γ and the severity of the neuropathological response has been reported in mice (Sternberg et al., 2005). This cytokine plays a central role in the mechanisms of pathology associated with experimental HAT meningoencephalitis, being involved in the initiation of the host immune response against trypanosomes and in the mechanisms of brain invasion (Kristensson et al., 2010; Mogk et al., 2017). Moreover, IFN-γ induces the release of CXCL10 in astrocytes and has been proposed to facilitate the passage of both lymphocytes and parasites into the brain parenchyma since mice deficient for CXCL10- or IFN-γ-receptor showed reduced brain infection (Masocha et al., 2004; Kristensson et al., 2010).

Our results suggest that MVs derived from S2 CSF have effects partially similar to those induced by IFN-γ on human astrocytes and could thus represent an important new player to consider in the understanding of the pathogenic mechanisms of HAT. Interestingly, astrocytes exposed to MVs-free CSF from the same HAT patients, did not show any relevant alteration in protein expression.

Although only a limited number of samples was investigated, our study shows, for the first time, the association of CSF-MVs with HAT late stage disease. Our observations pave the way for a number of investigations to elucidate the potential functional role of these MVs in HAT neuro-pathogenesis. These should include the evaluation of the effects of CSF-MVs on additional cell types—such as microglial cells, and the investigation of the potential presence of parasite-derived elements within the vesicle preparation (both as vesicle-like structures or soluble factors).

Finally, an in depth investigation of the molecular cargo of host-derived MVs might contribute to understand the mechanisms through which they act on target cells and might reveal novel potential disease biomarkers of clinically utility for both staging and treatment outcome evaluation.

## Data Availability Statement

The mass spectrometry proteomics data have been deposited to the ProteomeXchange Consortium via the PRIDE partner repository with the dataset identifier PXD014945.

## Ethics Statement

The studies involving human participants were reviewed and approved by Ministry of Health of the Democratic Republic of the Congo Commission for Medical Ethics of the Institute of Tropical Medicine Antwerp, Belgium. Written informed consent to participate in this study was provided by the participants or by a legal guardian/next of kin.

## Author Contributions

VD, J-CS, and NT conceived the study. VD and NT designed and performed experiments and performed data analyses. VL, DM, and PB provided the samples. NT wrote the manuscript. All authors read and revised the manuscript.

### Conflict of Interest

The authors declare that the research was conducted in the absence of any commercial or financial relationships that could be construed as a potential conflict of interest.
