# Supplementary material for: Cerebrospinal Fluid-Derived Microvesicles From Sleeping Sickness Patients Alter Protein Expression in Human Astrocytes
Source: Front Cell Infect Microbiol. 2019 Nov 20;9:391. doi: 10.3389/fcimb.2019.00391 (PMC6879452; doi:10.3389/fcimb.2019.00391)

Supplementary Material

Cerebrospinal Fluid-Derived Microvesicles from Sleeping Sickness Patients Alter Protein Expression in Human Astrocytes

Vito Dozio[1], Veerle Lejon[2], Dieudonné Mumba Ngoyi[3], Philippe Buscher[4], Jean-Charles Sanchez[1a]*, Natalia Tiberti[1,5a]*

Supplementary Table S1. Demographic description of the population analyzed by flow cytometry for microvesicle enumeration in CSF

	S1 (n=12)	Int (n=12)*	S2 (n=12)	p-value	test
**Gender (F), n**	8	5	5	ns	Chi square
**Age (years), mean ± SD**	39.1 ± 14.5	37.1 ± 14.0	35.8 ± 13.1	ns	Kruskal-Wallis
**WBC/µl in CSF, median [range]**	2 [1 - 4]	10 [2 - 18]	122 [12 - 447]	0.0001	Kruskal-Wallis[†]
**T^+ in CSF, n**	0	2	12	<0.0001	Chi square
**Presence of neurological signs, n**	3	8	11	0.003	Chi square

S1= Stage 1; Int= intermediate stage; S2=Stage 2; ns= non-significant, T^+ = trypanosomes in CSF
** Intermediate patients: CSF WBC ≤ 5/µl and T^+ (n=2); 5<WBC/µl ≤20 and T^- (n=10)*
† Dunn's pairwise comparison: S1 v. S2 < 0.0001; S1 vs. Int = 0.007; S2 vs. Int 0.003

Supplementary Table S2. Details of the antibodies and isotype controls used for the detection of MVs by flow cytometry

Target antigen	Clone	Isotype	Format	Conc. (μg/mL)
CD45	HI30	Mouse IgG1, k	APC	3
CD105	266	Mouse IgG1, k	PE	50
NSE	EPR3377	Rabbit, IgG	PE	500

Clone	Isotype	Format	Conc. (μg/mL)
MOPC-21	Mouse IgG1, k	APC	12.5
MOPC-21	Mouse IgG1, k	PE	50
EPR25A	Rabbit IgG	PE	500

Supplementary Table S3. Demographic description of the pools created for *in vitro* experiments

	Stage	Gender (F), n	Age, mean	T^{+} CSF, n	WBC/µl, mean	T-MVs*	$CD45^{+}$ MVs*
Pool 1	1	4	37.5	0	2	5024	513
Pool 2	1	0	34.5	0	2	2484	306
Pool 3	2	4	37.3	4	180.3	29094	7605
Pool 4	2	0	34.5	3	413.5	34231	6403
Pool 5	2	0	35.5	4	291	23015	4455
Pool 6	Int	4	36.8	2	4.8	3518	107
Pool 7	Int	0	38.0	0	12.5	5812	375

Each pool was obtained using CSF from n=4 patients. S1= Stage 1; Int= intermediate stage; S2=Stage 2; T^{+} *= trypanosomes in CSF*

** Number of MVs enriched from 2.4ml of CSF. Flow-cytometry measurements were performed on pelleted MVs.*

Supplementary Table S4. Additional regression analyses assessing the association between total-MVs (T-MVs) (S4A) or CD45$^+$L-MV (S4B) and HAT stage. Association between NSE$^+$N-MV (S4C) or CD105$^+$E-MV (S4D) and demographical and clinical variables.

S4A – Univariate regression for T-MVs

Variables	Intercept	[95% Confidence Interval]		p-value	model R-squared
**Stage**					
**1** (base)	0				
**2**	343.47	150.55	536.38	0.001	**0.422**
**Int.**	-99.70	-292.62	93.21	0.301	

S4B – Univariate regression for CD45$^+$L-MV

Variables	Intercept	[95% Confidence Interval]		p-value	model R-squared
**Stage**					
**1** (base)	0				
**2**	8.93	4.91	12.96	<0.0001	**0.434**
**Int.**	0.67	-3.36	4.69	0.737	

S4C - Regression analysis for NSE$^+$N-MVs

Variables	Intercept	[95% Confidence Interval]		p-value	model R-squared
Univariate					
**WBC/µL**	-0.01	-0.04	0.02	0.572	**0.009**
**Sex**	6.08	-1.64	13.81	0.119	**0.070**
**Age**	0.08	-0.22	0.38	0.576	**0.009**
**T+**	-3.12	-11.26	5.02	0.442	**0.018**
**NS+**	2.36	-5.81	10.54	0.561	**0.010**

S4D - Regression analysis for CD105$^+$E-MVs

Variables	Intercept	[95% Confidence Interval]		p-value	model R-squared
Univariate					
**WBC/µL**	-0.004	-0.02	0.01	0.511	**0.013**
**Sex**	-1.01	-3.98	1.96	0.495	**0.014**
**Age**	-0.03	-0.14	0.08	0.557	**0.010**
**T+**	0.99	-2.06	4.04	0.513	**0.013**
**NS+**	-0.66	-3.72	2.40	0.664	**0.006**

Table S5: Top-three biological networks significantly represented among proteins differentially abundant in the different study conditions vs. control.

Network	GO Process	Size	Target	p-Value
	**S1**			
**RPL8, Testin, RPS13, RPL13, RPL27**	Co-translational protein targeting to membrane, Protein localization to endoplasmic reticulum	50	7	2.23e-20
**H1FX, RPL7A, RPL23a, RPL39, RPL35**	Co-translational protein targeting to membrane, Protein localization to endoplasmic reticulum	50	6	3.10e-17
**Fibronectin, RPS6, FAK1, BETA-PIX, PtdIns(3,4,5)P3 intracellular**	Cell migration and motility	52	2	1.83e-05
	**INT**			
**RPL27, RPS2, RPL39, RPL34, MTCH2**	Co-translational protein targeting to membrane, Protein localization to endoplasmic reticulum	50	8	1.40e-22
**ALY, hnRNP A2, RALY, RPL36, RPL13A**	Viral transcription and gene expression	50	5	1.78e-13
**Fibronectin, FAK1, Paxillin, PAK1, CDC42**	Cell organization and motility<br>Neuron projection morphogenesis	61	1	9.67e-03
	**S2**			
**RPS30, Sideroflexin-3, RPS2, CREB1, RPS16**	Protein targeting to membrane	50	16	2.43e-46
**COX IV-1, RPS9, RPL13, Ubi-L, RPL36**	Actin cytoskeleton organization	50	10	8.03e-27
**PSF, REA, hnRNP L, hnRNP A1, RPL39**	Regulation of gene expression and metabolic processes	50	8	9.49e-21
	**IFN**			
**HLA-B39, HLA-B49, 1B59, 1B48, HLA-Cw12**	Antigen processing and presentation of endogenous peptide antigen via MHC class I via ER pathway, TAP-independent	50	44	1.87e-125
**Tapasin, TAP2 (PSF2), TAP1 (PSF1), Plakoglobin, HLA-Cw7**	Antigen processing and presentation of endogenous peptide antigen via MHC class I	50	19	1.21e-43
**STAT3, Shc, GRB2, FGFR1, c-Raf-1**	Cellular metabolism and signal transduction	52	1	5.55e-02

Supplementary Table S6. Proteomics results: comparison between cells exposed to S1 MVs vs. Int. MVs. Only proteins with a |FC| > 1.2 have been reported. Proteins significantly differentially expressed are highlighted in green.

			S1 MVs / Int. MVs	
**Protein AC**	**ID**	**Description**	**log2FC**	**p-value**
Q9BQ48	RM34	39S ribosomal protein L34, mitochondrial	-0.9611	0.8229
Q01581	HMCS1	Hydroxymethylglutaryl-CoA synthase, cytoplasmic	-0.8659	0.7739
Q99570	PI3R4	Phosphoinositide 3-kinase regulatory subunit 4	-0.7409	0.9694
Q9NUQ9	FA49B	Protein FAM49B	-0.6680	0.9747
L0R6Q1	S35U4	SLC35A4 upstream open reading frame protein	-0.6643	0.1758
P05161	ISG15	Ubiquitin-like protein ISG15	-0.6483	0.9670
O95864	FADS2	Fatty acid desaturase 2	-0.5869	0.5684
Q5TAX3	TUT4	Terminal uridylyltransferase 4	-0.5646	0.9135
Q5SY85	F201A	Protein FAM201A	-0.5505	0.9500
**P02452**	**CO1A1**	**Collagen alpha-1(I) chain**	**-0.5418**	**0.0001**
Q9Y6D5	BIG2	Brefeldin A-inhibited guanine nucleotide-exchange protein 2	-0.5233	0.9893
O95104	SFR15	Splicing factor, arginine/serine-rich 15	-0.5114	0.9573
Q5TFE4	NT5D1	5'-nucleotidase domain-containing protein 1	-0.4977	0.9561
P98082	DAB2	Disabled homolog 2	-0.4876	0.6734
Q86VR2	RETR3	Reticulophagy regulator 3	-0.4832	0.9481
Q9NPA8	ENY2	Transcription and mRNA export factor ENY2	-0.4787	0.9115
P60468	SC61B	Protein transport protein Sec61 subunit beta	-0.4725	0.9839
Q96RE7	NACC1	Nucleus accumbens-associated protein 1	-0.4523	0.7545
Q9NYY8	FAKD2	FAST kinase domain-containing protein 2, mitochondrial	-0.4232	0.8662
P35527	K1C9	Keratin, type I cytoskeletal 9	-0.4174	0.9940
P09110	THIK	3-ketoacyl-CoA thiolase, peroxisomal	-0.4132	0.9810
Q96LC7	SIG10	Sialic acid-binding Ig-like lectin 10	-0.4074	0.9782
P14927	QCR7	Cytochrome b-c1 complex subunit 7	-0.4034	0.4810
Q96SU4	OSBL9	Oxysterol-binding protein-related protein 9	-0.4026	0.7597
Q9NZN5	ARHGC	Rho guanine nucleotide exchange factor 12	-0.4023	0.9197
Q99996	AKAP9	A-kinase anchor protein 9	-0.4017	0.8918
Q13620	CUL4B	Cullin-4B	-0.3976	0.9781
Q6P587	FAHD1	Acylpyruvase FAHD1, mitochondrial	-0.3901	0.9758
Q9HAV7	GRPE1	GrpE protein homolog 1, mitochondrial	-0.3890	0.9122
O60518	RNBP6	Ran-binding protein 6	-0.3847	0.6587
Q96GX5	GWL	Serine/threonine-protein kinase greatwall	-0.3724	0.8158
Q9Y4F1	FARP1	FERM, RhoGEF and pleckstrin domain-containing protein 1	-0.3703	0.6122
Q96EY8	MMAB	Cob(I)yrinic acid a,c-diamide adenosyltransferase, mitochondrial	-0.3696	0.9562
Q14116	IL18	Interleukin-18	-0.3666	0.6869
Q6ZMZ3	SYNE3	Nesprin-3	-0.3557	0.9494
P10301	RRAS	Ras-related protein R-Ras	-0.3538	0.9772
Q8WTW3	COG1	Conserved oligomeric Golgi complex subunit 1	-0.3520	0.8534
Q9NRX4	PHP14	14 kDa phosphohistidine phosphatase	-0.3504	0.9793
Q9Y6M7	S4A7	Sodium bicarbonate cotransporter 3	-0.3469	0.9812
Q96K76	UBP47	Ubiquitin carboxyl-terminal hydrolase 47	-0.3436	0.9069
Q9C035	TRIM5	Tripartite motif-containing protein 5	-0.3395	0.9646
P14550	AK1A1	Alcohol dehydrogenase [NADP(+)]	-0.3384	0.8984
Q9UDW1	QCR9	Cytochrome b-c1 complex subunit 9	-0.3293	0.9213
Q9UBL3	ASH2L	Set1/Ash2 histone methyltransferase complex subunit ASH2	-0.3289	0.9461
Q92575	UBXN4	UBX domain-containing protein 4	-0.3289	0.9899
O75718	CRTAP	Cartilage-associated protein	-0.3285	0.7905

Q9BQE5	APOL2	Apolipoprotein L2	-0.3209	0.7180
P52434	RPAB3	DNA-directed RNA polymerases I, II, and III subunit RPABC3	-0.3166	0.9903
Q9Y3E5	PTH2	Peptidyl-tRNA hydrolase 2, mitochondrial	-0.3132	0.9839
Q2TAA2	IAH1	Isoamyl acetate-hydrolyzing esterase 1 homolog	-0.3121	0.7943
Q6IAA8	LTOR1	Ragulator complex protein LAMTOR1	-0.3038	0.9720
Q8WVM8	SCFD1	Sec1 family domain-containing protein 1	-0.2987	0.9108
Q96SI9	STRBP	Spermatid perinuclear RNA-binding protein	-0.2982	0.8424
Q96FN4	CPNE2	Copine-2	-0.2966	0.9579
Q5VT52	RPRD2	Regulation of nuclear pre-mRNA domain-containing protein 2	-0.2965	0.9646
Q9BSV6	SEN34	tRNA-splicing endonuclease subunit Sen34	-0.2960	0.9660
Q9Y5Z4	HEBP2	Heme-binding protein 2	-0.2937	0.9846
Q9UBC2	EP15R	Epidermal growth factor receptor substrate 15-like 1	-0.2919	0.8819
Q8TDZ2	MICA1	[F-actin]-methionine sulfoxide oxidase MICAL1	-0.2878	0.9367
Q9P013	CWC15	Spliceosome-associated protein CWC15 homolog	-0.2861	0.8794
P80303	NUCB2	Nucleobindin-2	-0.2860	0.9271
Q14697	GANAB	Neutral alpha-glucosidase AB	-0.2827	0.9905
Q92905	CSN5	COP9 signalosome complex subunit 5	-0.2815	0.9872
Q9HB19	PKHA2	Pleckstrin homology domain-containing family A member 2	-0.2809	0.8173
P53803	RPAB4	DNA-directed RNA polymerases I, II, and III subunit RPABC4	-0.2809	0.9241
Q8NCF5	NF2IP	NFATC2-interacting protein	-0.2788	0.9635
P27635	RL10	60S ribosomal protein L10	-0.2778	0.9799
Q9GZU8	F192A	Protein FAM192A	-0.2772	0.9511
Q15276	RABE1	Rab GTPase-binding effector protein 1	-0.2771	0.7033
P46013	KI67	Proliferation marker protein Ki-67	-0.2746	0.8597
**O15460**	**P4HA2**	**Prolyl 4-hydroxylase subunit alpha-2**	**-0.2722**	**0.0432**
Q8TDY2	RBCC1	RB1-inducible coiled-coil protein 1	-0.2699	0.9709
Q15084	PDIA6	Protein disulfide-isomerase A6	-0.2683	0.8630
P53634	CATC	Dipeptidyl peptidase 1	-0.2679	0.9604
P39880	CUX1	Homeobox protein cut-like 1	-0.2677	0.8859
P14618	KPYM	Pyruvate kinase PKM	-0.2673	0.9930
Q96M27	PRRC1	Protein PRRC1	-0.2659	0.9065
O76054	S14L2	SEC14-like protein 2	-0.2650	0.9780
O75530	EED	Polycomb protein EED	-0.2645	0.8605
P37268	FDFT	Squalene synthase	-0.2636	0.9641
Q86X55	CARM1	Histone-arginine methyltransferase CARM1	0.2613	0.9802
Q96C90	PP14B	Protein phosphatase 1 regulatory subunit 14B	0.2623	0.9062
O75306	NDUS2	NADH dehydrogenase [ubiquinone] iron-sulfur protein 2, mitochondrial	0.2629	0.3707
**P21796**	**VDAC1**	**Voltage-dependent anion-selective channel protein 1**	**0.2672**	**0.0012**
**P23246**	**SFPQ**	**Splicing factor, proline- and glutamine-rich**	**0.2753**	**0.0037**
O60282	KIF5C	Kinesin heavy chain isoform 5C	0.2762	0.8997
O75380	NDUS6	NADH dehydrogenase [ubiquinone] iron-sulfur protein 6, mitochondrial	0.2885	0.2807
**Q99623**	**PHB2**	**Prohibitin-2**	**0.2926**	**0.0000**
Q12800	TFCP2	Alpha-globin transcription factor CP2	0.2929	0.9860
Q8IVD9	NUDC3	NudC domain-containing protein 3	0.2933	0.8938
Q07021	C1QBP	Complement component 1 Q subcomponent-binding protein, mitochondrial	0.2935	0.8269
P09104	ENOG	Gamma-enolase	0.2949	0.9514
**P45880**	**VDAC2**	**Voltage-dependent anion-selective channel protein 2**	**0.2959**	**0.0005**
Q9BUF5	TBB6	Tubulin beta-6 chain	0.3072	0.9915
Q6PD74	AAGAB	Alpha- and gamma-adaptin-binding protein p34	0.3099	0.9842
P48426	PI42A	Phosphatidylinositol 5-phosphate 4-kinase type-2 alpha	0.3103	0.9465
P82921	RT21	28S ribosomal protein S21, mitochondrial	0.3108	0.8356

Q9P1Z2	CACO1	Calcium-binding and coiled-coil domain-containing protein 1	0.3127	0.9355
Q8NEW0	ZNT7	Zinc transporter 7	0.3153	0.8721
P13995	MTDC	Bifunctional methylenetetrahydrofolate dehydrogenase/cyclohydrolase, mitochondrial	0.3259	0.9050
O75843	AP1G2	AP-1 complex subunit gamma-like 2	0.3267	0.8952
Q15735	PI5PA	Phosphatidylinositol 4,5-bisphosphate 5-phosphatase A	0.3285	0.8282
O75381	PEX14	Peroxisomal membrane protein PEX14	0.3295	0.7979
Q86SE5;	RALYL	RNA-binding Raly-like protein	0.3323	0.7646
Q9Y6C9	MTCH2	Mitochondrial carrier homolog 2	0.3328	0.4444
Q16795	NDUA9	NADH dehydrogenase [ubiquinone] 1 alpha subcomplex subunit 9, mitochondrial	0.3469	0.7344
**O94901**	**SUN1**	**SUN domain-containing protein 1**	**0.3678**	**0.0017**
O75367	H2AY	Core histone macro-H2A.1	0.3825	0.3007
P11166	GTR1	Solute carrier family 2, facilitated glucose transporter member 1	0.3913	0.9927
O95182	NDUA7	NADH dehydrogenase [ubiquinone] 1 alpha subcomplex subunit 7	0.3953	0.5601
P68871	HBB	Hemoglobin subunit beta	0.4317	0.9084
Q9P0J0	NDUAD	NADH dehydrogenase [ubiquinone] 1 alpha subcomplex subunit 13	0.4469	0.9364
P41567	EIF1	Eukaryotic translation initiation factor 1	0.4489	0.7571
Q6QHK4	FIGLA	Factor in the germline alpha	0.5507	0.8788
P04004	VTNC	Vitronectin	0.6044	0.1517
Q05655	KPCD	Protein kinase C delta type	0.6730	0.3196
P02768	ALBU	Serum albumin	0.8032	0.8692
P0CG39	POTEJ	POTE ankyrin domain family member J	0.9907	0.9545
P0CG12	CTF8A	Chromosome transmission fidelity protein 8 homolog isoform 2	2.3229	0.9480

Supplementary Table S7. Proteomics results: comparison between cells exposed to S2 MVs vs. Int. MVs. Only proteins with a |FC| > 1.2 have been reported.

			S2 MVs / Int. MVs	
**Protein AC**	**ID**	**Description**	**log2FC**	**p-value**
P15153	RAC2	Ras-related C3 botulinum toxin substrate 2	-1.5534	0.9153
P35527	K1C9	Keratin, type I cytoskeletal 9	-1.2559	0.9772
Q5T0F9	C2D1B	Coiled-coil and C2 domain-containing protein 1B	-1.1159	0.9816
Q5TAX3	TUT4	Terminal uridylyltransferase 4	-0.6956	0.8562
Q92575	UBXN4	UBX domain-containing protein 4	-0.6721	0.9757
Q96LC7	SIG10	Sialic acid-binding Ig-like lectin 10	-0.6292	0.9463
Q9C035	TRIM5	Tripartite motif-containing protein 5	-0.6107	0.9762
Q9Y6D5	BIG2	Brefeldin A-inhibited guanine nucleotide-exchange protein 2	-0.5578	0.9883
Q9HAV7	GRPE1	GrpE protein homolog 1, mitochondrial	-0.5254	0.7463
P06899	H2B1J	Histone H2B type 1-J	-0.5109	0.9720
P52434	RPAB3	DNA-directed RNA polymerases I, II, and III subunit RPABC3	-0.4696	0.9905
Q8TDY2	RBCC1	RB1-inducible coiled-coil protein 1	-0.4418	0.8775
Q9Y3E0	GOT1B	Vesicle transport protein GOT1B	-0.4362	0.9575
P04264	K2C1	Keratin, type II cytoskeletal 1	-0.3837	0.9954
P35908	K22E	Keratin, type II cytoskeletal 2 epidermal	-0.3784	0.9948
L0R6Q1	S35U4	SLC35A4 upstream open reading frame protein	-0.3697	0.8728
Q9NYY8	FAKD2	FAST kinase domain-containing protein 2, mitochondrial	-0.3561	0.9208
Q9UBL3	ASH2L	Set1/Ash2 histone methyltransferase complex subunit ASH2	-0.3350	0.9856
Q9BQ48	RM34	39S ribosomal protein L34, mitochondrial	-0.3224	0.9915
P53803	RPAB4	DNA-directed RNA polymerases I, II, and III subunit RPABC4	-0.3210	0.9679
Q01581	HMCS1	Hydroxymethylglutaryl-CoA synthase, cytoplasmic	-0.3200	0.9828
O75843	AP1G2	AP-1 complex subunit gamma-like 2	-0.3074	0.9903
Q9BYT8	NEUL	Neurolysin, mitochondrial	-0.3012	0.9943
Q9Y6M7	S4A7	Sodium bicarbonate cotransporter 3	-0.2855	0.9846
Q15906	VPS72	Vacuolar protein sorting-associated protein 72 homolog	-0.2776	0.9034
Q9HC84	MUC5B	Mucin-5B	-0.2759	0.9611
Q9Y4F1	FARP1	FERM, RhoGEF and pleckstrin domain-containing protein 1	-0.2754	0.8588
Q9NZN5	ARHGC	Rho guanine nucleotide exchange factor 12	-0.2754	0.8685
Q9NRX4	PHP14	14 kDa phosphohistidine phosphatase	-0.2749	0.9908
O94992	HEXI1	Protein HEXIM1	-0.2737	0.8525
Q9UHN1	DPOG2	DNA polymerase subunit gamma-2, mitochondrial	-0.2730	0.9801
Q96A11	G3ST3	Galactose-3-O-sulfotransferase 3	-0.2711	0.8515
Q96EY8	MMAB	Cob(I)yrinic acid a,c-diamide adenosyltransferase, mitochondrial	-0.2694	0.9784
Q8TDZ2	MICA1	[F-actin]-methionine sulfoxide oxidase MICAL1	-0.2610	0.9936
P36578	RL4	60S ribosomal protein L4	0.2631	0.7717
P30041	PRDX6	Peroxiredoxin-6	0.2656	0.9839
Q7LBC6	KDM3B	Lysine-specific demethylase 3B	0.2722	0.9087
P68371	TBB4B	Tubulin beta-4B chain	0.2767	0.9881
P61619	S61A1	Protein transport protein Sec61 subunit alpha isoform 1	0.2774	0.9936
Q12800	TFCP2	Alpha-globin transcription factor CP2	0.2816	0.9845
Q13144	EI2BE	Translation initiation factor eIF-2B subunit epsilon	0.2887	0.9492
A6NKD9	CC85C	Coiled-coil domain-containing protein 85C	0.2897	0.9622
P61313	RL15	60S ribosomal protein L15	0.2918	0.7866
Q9UMS0	NFU1	NFU1 iron-sulfur cluster scaffold homolog, mitochondrial	0.3048	0.7670
Q96KN7	RPGR1	X-linked retinitis pigmentosa GTPase regulator-interacting protein 1	0.3279	0.8466
Q8TCS8	PNPT1	Polyribonucleotide nucleotidyltransferase 1, mitochondrial	0.3664	0.9450

Q6PD74	AAGAB	Alpha- and gamma-adaptin-binding protein p34	0.3758	0.9776
Q8NEW0	ZNT7	Zinc transporter 7	0.3955	0.8307
Q9BUF5	TBB6	Tubulin beta-6 chain	0.4724	0.9677
P11166	GTR1	Solute carrier family 2, facilitated glucose transporter member 1	0.4752	0.9887
Q05655	KPCD	Protein kinase C delta type	0.4790	0.6250
Q9HAU0	PKHA5	Pleckstrin homology domain-containing family A member 5	0.4873	0.9431
Q9GZT9	EGLN1	Egl nine homolog 1	0.6299	0.7783
P0CG39	POTEJ	POTE ankyrin domain family member J	1.0352	0.9324
P61626	LYSC	Lysozyme C	1.1486	0.9042
P02768	ALBU	Serum albumin	1.1588	0.5137
P0CG12	CTF8A	Chromosome transmission fidelity protein 8 homolog isoform 2	1.4530	0.9827
Q9HAU5	RENT2	Regulator of nonsense transcripts 2	2.2976	0.9791

Supplementary Figure S1. Representative scatter plots of the results obtained by flow cytometry for the detection of CSF MVs. MV gate was defined based on positivity at CTV or AnV labelling. MV gate= microvesicle gate; Beads= FlowCount bead region; CTV= CellTrace™ Violet; AnV FITC= Annexin AV fluorescein isothiocyanate; NSE-PE= neuron specific enolase – phycoerythrin; CD105-PE= CD105- phycoerythrin; CD45-APC= CD45-allophycocyanin. Images obtained with FlowJo™ 10.6.1.

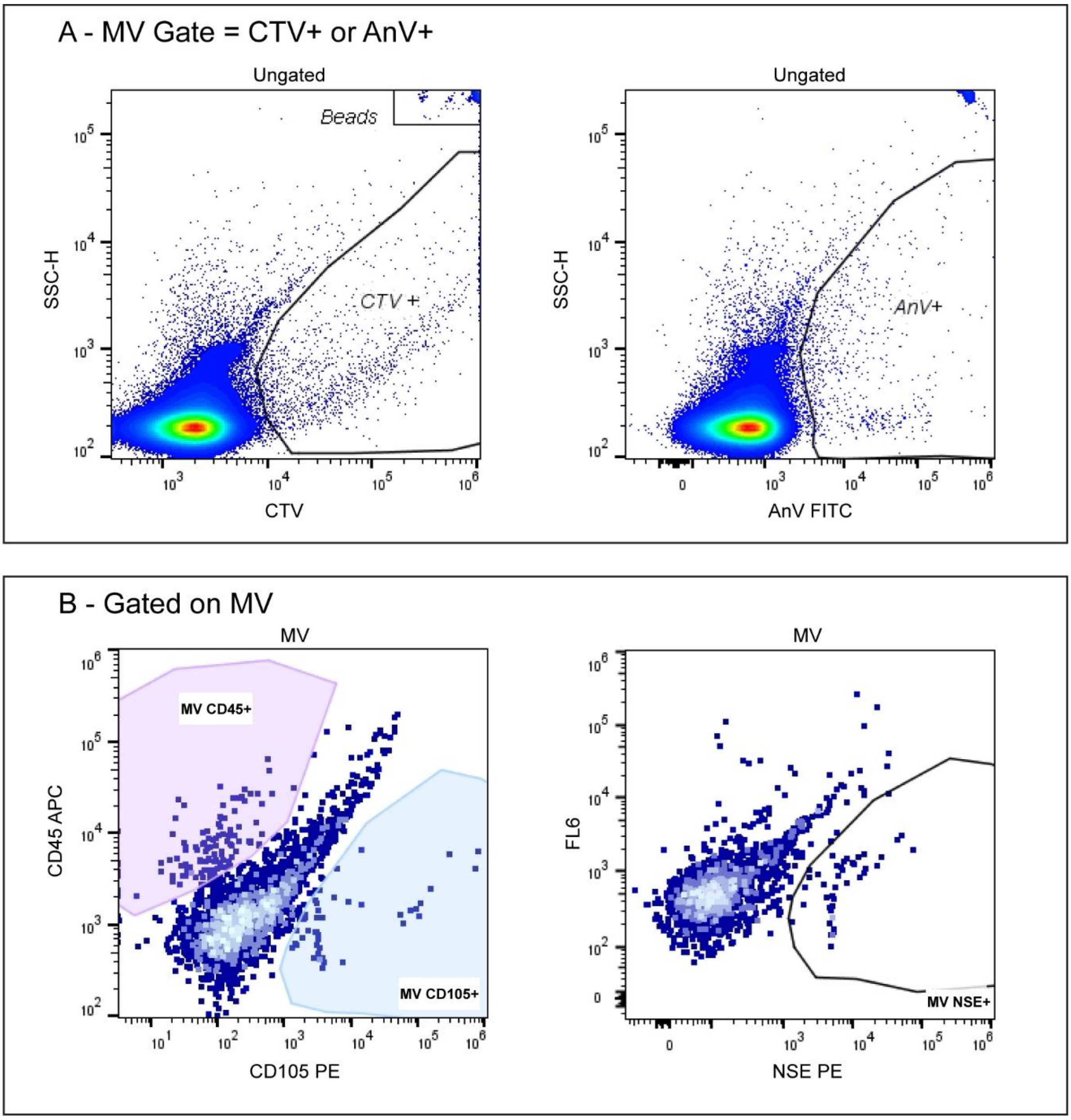

Supplementary Figure S2. Results obtained upon exposure of human astrocytes to MV-free CSF. (A) Comparison of the proteins identified in the experiment performed with MVs (Exp.1) and with MV-free CSF (Exp.2). (**B)** Proteins differentially abundant. The number of proteins differentially abundant upon exposure of human astrocytes to IFN-γ in the two experiments are reported. The number of proteins differentially abundant upon astrocyte exposure to either HAT MVs or MV-free HAT CSF is also shown.

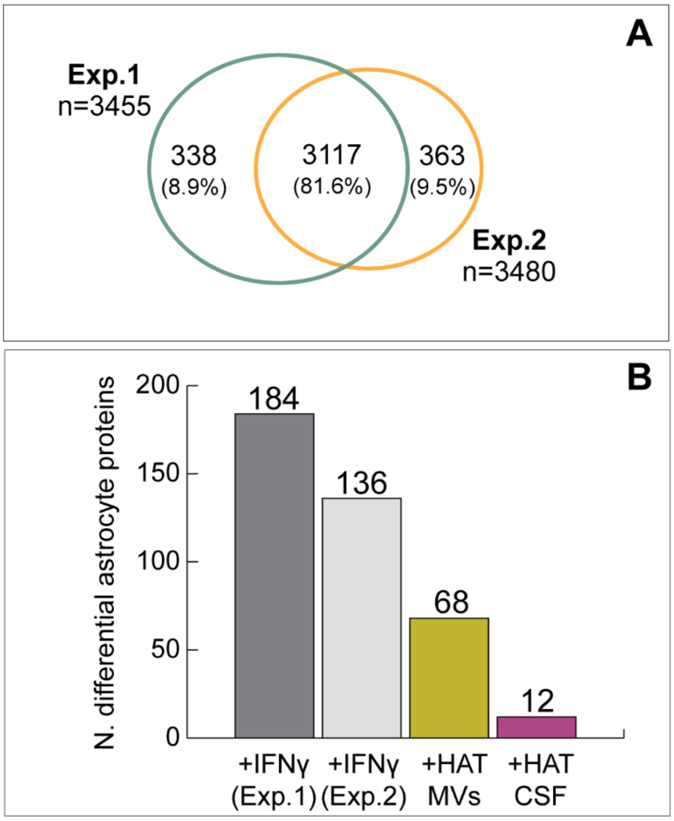

Supplement: Supplementary file 2 [file Data_Sheet_1.PDF]
